# Discovered by genomics: putative reductive dehalogenases with N-terminus transmembrane helixes

**DOI:** 10.1093/femsec/fiz048

**Published:** 2019-04-03

**Authors:** Siavash Atashgahi

**Affiliations:** 1 Laboratory of Microbiology, Wageningen University & Research, Stippeneng 4, 6708 WE Wageningen, The Netherlands; 2 Department of Microbiology, IWWR, Radboud University Nijmegen, Heyendaalseweg 135, 6525 AJ Nijmegen, The Netherlands; 3 Soehngen Institute of Anaerobic Microbiology, Heyendaalseweg 135, 6525 AJ Nijmegen, The Netherlands

**Keywords:** reductive dehalogenase, organohalide respiration, transmembrane helix

## Abstract

Attempts for bioremediation of toxic organohalogens resulted in the identification of organohalide-respiring bacteria harbouring reductive dehalogenases (RDases) enzymes. RDases consist of the catalytic subunit (RdhA, encoded by *rdhA*) that does not have membrane-integral domains, and a small putative membrane anchor (RdhB, encoded by *rdhB*) that (presumably) locates the A subunit to the outside of the cytoplasmic membrane. Recent genomic studies identified a putative *rdh* gene in an uncultured deltaproteobacterial genome that was not accompanied by an *rdhB* gene, but contained transmembrane helixes in N-terminus. Therefore, rather than having a separate membrane anchor protein, this putative RDase is likely a hybrid of RdhA and RdhB, and directly connected to the membrane with transmembrane helixes. However, functionality of the hybrid putative RDase remains unknown. Further analysis showed that the hybrid putative *rdh* genes are present in the genomes of pure cultures and uncultured members of Bacteriodetes and Deltaproteobacteria, but also in the genomes of the candidate divisions. The encoded hybrid putative RDases have cytoplasmic or exoplasmic C-terminus localization, and cluster phylogenetically separately from the existing RDase groups. With increasing availability of (meta)genomes, more diverse and likely novel *rdh* genes are expected, but questions regarding their functionality and ecological roles remain open.

## Teaser

Recent genomic analysis revealed putative reductive dehalogenase genes from extreme subsurface environments that unlike known reductive dehalogenases have membrane integral domains.

## INTRODUCTION

With the advent of the Industrial Revolution, human impacts on the environment increased dramatically. Hazardous halogenated organic compounds, organohalogens, were widely distributed in the natural environment through careless use and indiscriminate disposal, and caused major public concerns due to possible effects on human and environmental health (Häggblom 1992). In attempts for organohalogen bioremediation, a hallmark discovery was the identification of microbes that could use organohalogens as electron acceptors and reductively dehalogenate them (Suflita *et al*. 1982). This new metabolism, later termed organohalide respiration (OHR), has found great practical application in bioremediation. Accordingly, bioaugmentation with microbial consortia containing organohalide-respiring bacteria (OHRB) has become a showcase of successful engineered remediation of contaminated environments (Ellis *et al*. 2000; Stroo, Leeson and Ward 2012).

Over the past three decades, a wealth of knowledge has been obtained about the ecophysiology, biochemistry and environmental distribution of OHRB (Häggblom and Bossert 2003; Adrian and Löffler 2016). Using biochemical, PCR-based and (meta)genomic analysis, reductive dehalogenases (RDases) have been identified as the key enzymes of OHR (Lu *et al*. 2015; Hug 2016). The RDase-encoding genes (*rdh*) have a conserved operon structure that consists of *rdhA*, coding for the catalytic subunit (RdhA); *rdhB*, coding for a small putative membrane anchor (RdhB) that (presumably) locates the A subunit to the outside of the cytoplasmic membrane; and a variable set of accessory genes (e.g. *rdhCTKZED*) (Kruse, Smidt and Lechner 2016). The catalytic subunits (RdhAs) are characterized by two iron-sulfur clusters (FeS1: CXXCXXCXXXCP; FeS2: CXXCXXXCP) and an N-terminus twin-arginine translocation motif (TAT: RRXFXK) (Holliger, Wohlfarth and Diekert 1998). This signal peptide is necessary for secretion of the mature RdhA protein through the cell membrane to the outer side of the cytoplasmic membrane (Smidt and de Vos 2004).

A second type of *rdhA* genes were discovered that lacked TAT motif, were located in the cytoplasm, and lacked respiratory function. This group was termed as ‘catabolic’ reductive dehalogenase that are used to convert organohalogens to non-halogenated compounds to be used as carbon sources (Chen *et al*. 2013; Payne *et al*. 2015). These types of *rdhA* genes were mostly found in marine than terrestrial environments (Reviewed in Atashgahi, Häggblom and Smidt 2018a).

### Putative *rdh* genes with N-terminus transmembrane helixes

**Figure 1.**
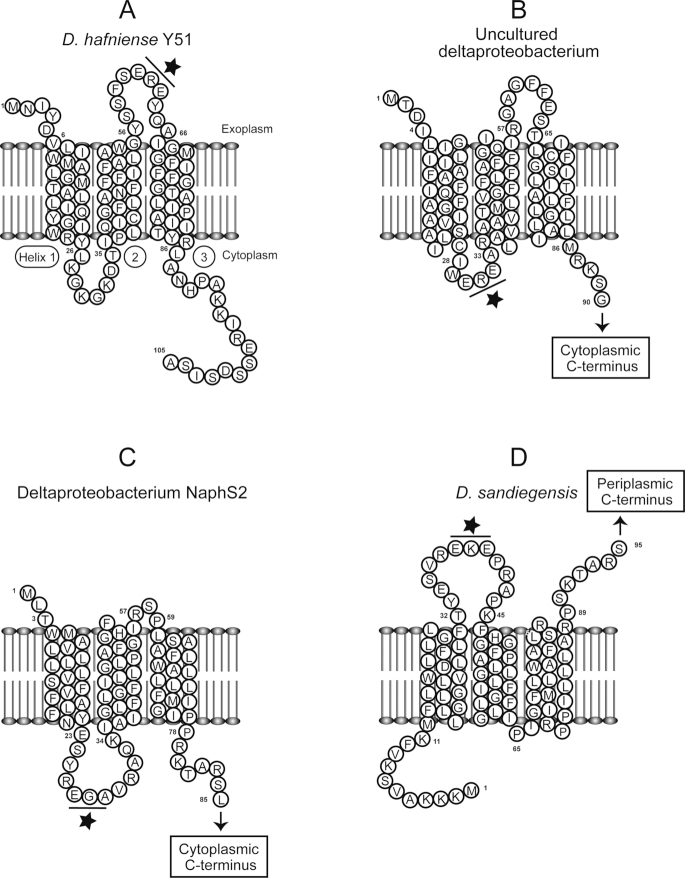
Predicted topology of the PceB protein of *D. hafniense* Y51 **(A)**, and N-terminus TMHs of the hybrid putative RDases from uncultured deltaproteobacterium (SAG2) obtained from the Aarhus Bay **(B)**, deltaproteobacterium strain NaphS2 **(C)**, and *D. sandiegensis***(D)**. The position of the EXE motif is indicated by a star. Note that in panel B, C and D, only partial sequences of the hybrid putative RDases containing N-terminus TMHs were shown. TMHs were detected using TMHMM Server v. 2.0 (Sonnhammer, Von Heijne and Krogh 1998). Permission to reprint panel A was obtained from (Schubert *et al*. 2018).

A recent single-cell genomic study from marine sediments in the Aarhus Bay discovered a third type of potential RDases in uncultured *Desulfatiglans*-related deltaproteobacterium (Jochum *et al*. 2018). A single-cell genome (SAG2) contained a putative *rdh* gene that is not accompanied by an *rdhB*, does not encode a TAT signal peptide, and as a unique feature, encodes three transmembrane helices (TMHs) in the N-terminus. Whereas the known respiratory RDases do not have membrane-integral domains, most RdhBs have three TMHs (Fig. 1). For instance, similar to the RdhB of *Desulfitobacterium hafniense* Y51 (Fig. 1A), the putative RDase from the uncultured *Desulfatiglans*-related deltaproteobacterium (Fig. 1B) has an exoplasmic N-terminus, followed by three TMHs. The remaining C-terminus contains the two binding motifs for FeS clusters, features of the known RDases. However, as the possible catalytic site, the C-terminus is facing the inner side of the cytoplasmic membrane (Fig. 1B) which is a likely localization in absence of the TAT signal peptide. The short cytoplasmic loop between helix 1 and 2 contains the two conserved glutamic acid residues (EXE motif) (Fig. 1B), proposed to play a role in the RdhA–RdhB interaction (Schubert *et al*. 2018). Similar cytoplasmic localization of the C-terminus of the putative RDase may enable such an interaction with this loop. Therefore, rather than having a separate membrane anchor protein, this putative RDase is predicted to act like a hybrid of RdhB and RdhA, and likely directly connected to the membrane with the TMHs.

The study of Jochum *et al*. further revealed that the hybrid putative *rdh* is similar to the putative *rdh* of two deltaproteobacterial pure cultures, i.e. deltaproteobacterium strain NaphS2 and *Dethiosulfatarculus sandiegensis* (Jochum *et al*. 2018). Indeed the putative *rdh* genes of these bacteria are not accompanied by an *rdhB* gene, lack TAT motif and contain three N-terminus TMHs. Similar to the putative RDase of the uncultured *Desulfatiglans*-related proteobacterium obtained from the Aarhus Bay (Fig. 1B), the putative RDase of the strain NaphS2 (Fig. 1C) has cytoplasmic C-terminus. In contrast, the putative RDase of *D. sandiegensis* has exoplasmic C-terminus (Fig. 1D), similar to the known RDases. The EXE motif in the loop between helix 1 and 2 is facing exoplasm, enabling potential interactions with the exoplasmic C-terminus (Fig. 1D). The three putative RDase share 46%–58% amino acid identity to each other, but share lower identity to the known RDases, e.g. 26%–29% identity to the TceA of *Dehalococcoides mccartyi* strain195 (DET0079). Although the existence of *rdh* genes lacking the TAT motif and *rdhB* were reported in the genomes of strain NaphS2 and *D. sandiegensis* (Sanford, Chowdhary and Löffler 2016; Liu and Häggblom 2018), the existence of TMHs in their putative RDase proteins were not reported. However, functionality of the hybrid putative RDases remains unknown.

### The hybrid putative *rdh* genes are widespread

**Figure 2.**
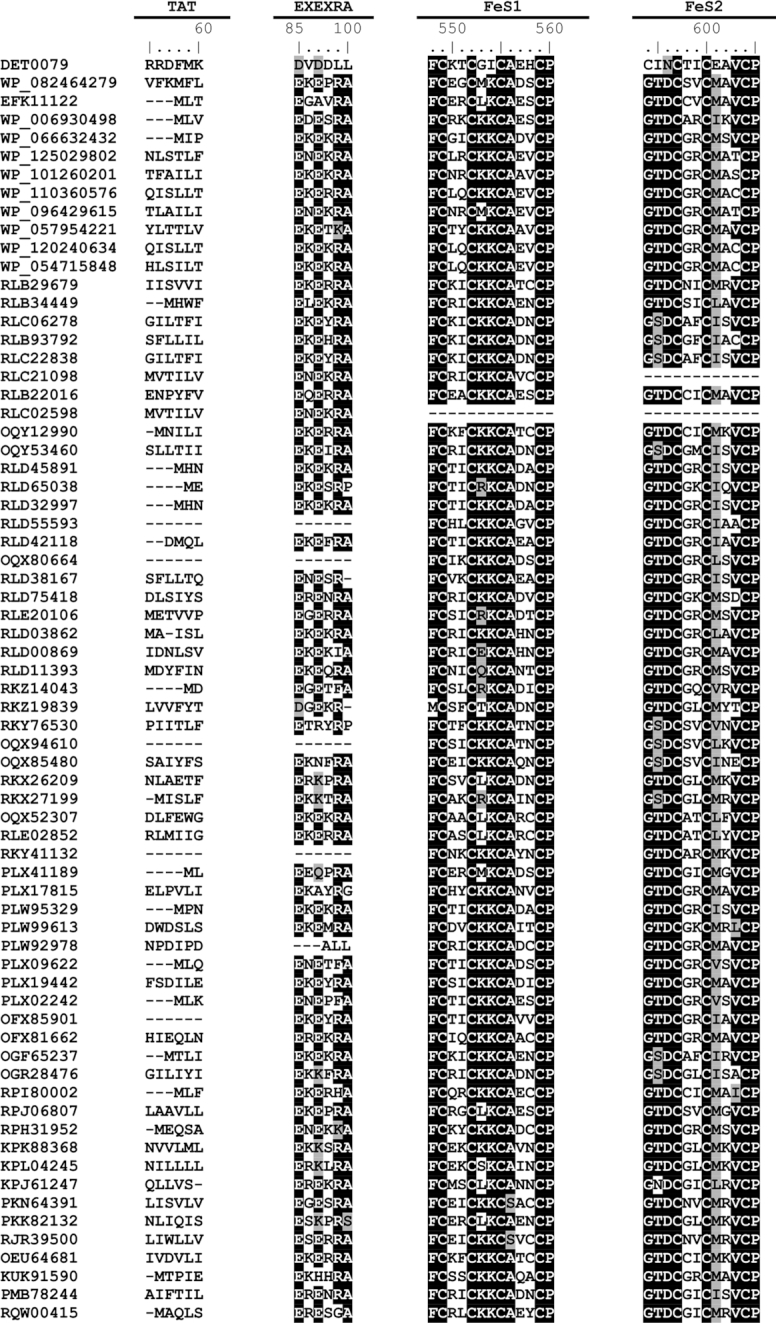
Sequence alignment of the hybrid putative RDases. Only conserved sequence motifs among experimentally characterized RDases (TAT, FeS1, FeS2), and the conserved glutamic acid residues (EXE) are included. The accession numbers are ordered according to Table 1, except the first accession number that belongs to TceA of *Dehalococcoides mccartyi* strain 195. ClustalW (Thompson, Higgins and Gibson 1994) multiple sequence alignment was conducted using BioEdit version 7.2.5 (http:/bioedit.software.informer.com/).

The sequence of the putative RDase of the uncultured proteobacterium obtained from the Aarhus Bay (Jochum *et al*. 2018) was used as a query in blastp searches against the NCBI non-redundant protein database in December 2018. The results showed that beyond the three identified proteobacterial hybrid putative *rdh* (Jochum *et al*. 2018), many other similar genes exist in the genomes of pure cultures as well as metagenome-assembled genomes (MAGs) that have gone unrecognized so far (Table 1). The majority of the sequences have three TMHs (detected using TMHMM Server v. 2.0 (Sonnhammer, Von Heijne and Krogh 1998)), the EXE motifs in their N-terminus, and either cytoplasmic or exoplasmic C-terminus containing the two FeS motifs (Table 1, Fig. 2). The C1–C5 regions from known the RDases are also conserved among the hybrid putative RDases (Fig. S1, Supporting Information), however, they are clustered phylogenetically separately from the existing RDase groups (Hug *et al*. 2013; Hug 2016) (Fig. S2, Supporting Information). Notably, the majority of the putative RDases are annotated as hypothetical proteins during automated annotation of the genomes.

**Table 1.** List of the hybrid putative RDases with TMHs in their N-terminus. Sequence information and the predicted functions by the automated annotation for each sequence are included in Supporting Information.

Organism	Length (aa)	TMH	C-terminus orientation	GenBank accession number	Sample source used for (meta)genome sequencing	Reference
Deltaproteobacteria bacterium	482	3	Cytoplasmic	- ^a^	Marine sediment from Aarhus Bay	(Jochum *et al*. 2018)
*Dethiosulfatarculus sandiegensis*	487	3	Exoplasmic	WP_08 246 4279	Pure deltaproteobacterial culture isolated from a methanogenic long-chain paraffins degrading consortium obtained from marine sediments	(Davidova *et al*. 2016)
Deltaproteobacterium NaphS2	478	3	Cytoplasmic	EFK11122	Pure deltaproteobacterial culture isolated from naphthalene-degrading enrichment obtained from marine sediments	(Galushko *et al*. 1999; Didonato Jr *et al*. 2010)
Marinifilaceae bacterium strain SPP2	459	3	Exoplasmic	WP_09 642 9615	Pure Marinilabiliales culture isolated from the Antarctic marine sediment	(Watanabe, Kojima and Fukui 2018)
*Marinifilum fragile*	456	3	Exoplasmic	WP_05 471 5848	Pure Marinilabiliales culture isolated from tidal flat sediment in Korea	(Na *et al*. 2009)
*Marinifilum breve*	457	3	Cytoplasmic	WP_110 360 576	Pure Marinilabiliales culture isolated from the Yongle Blue Hole in the South China Sea	(Fu *et al*. 2018)
*Marinifilum flexuosum*	454	3	Cytoplasmic	WP_120 240 634	Pure Marinilabiliales culture isolated from coastal Mediterranean Sea water	(Ruvira *et al*. 2013)
Ancylomarina sp. M1P	450	3	Cytoplasmic	WP_125 029 802	Pure Marinilabiliales culture isolated from Black Sea water	Unpublished
*Labilibaculum filiforme*	454	3	Exoplasmic	WP_101 260 201	Pure Marinilabiliales culture isolated from the subsurface sediments of the Baltic Sea	(Vandieken *et al*. 2018)
*Labilibacter marinus*	444	3	Cytoplasmic	WP_06 663 2432	Pure Marinilabiliales culture isolated from marine sediment at Weihai in China	(Liu *et al*. 2015; Lu *et al*. 2017)
*Salinivirga cyanobacteriivorans*	453	3	Cytoplasmic	WP_05 795 4221	Pure Marinilabiliales culture isolated from the suboxic zone of a hypersaline cyanobacterial mat	(Ben Hania *et al*. 2017)
*Caldithrix abyssi*	444	3	Cytoplasmic	WP_0 069 30498	Pure Calditrichales culture isolated from Mid-Atlantic Ridge hydrothermal vent	(Miroshnichenko *et al*. 2003; Kublanov *et al*. 2017)
Deltaproteobacteria bacterium	491	3	Exoplasmic	RLB29679	Hydrothermal sediments	(Dombrowski, Teske and Baker 2018)
Deltaproteobacteria bacterium	451	3	Exoplasmic	RLB34449	Hydrothermal sediments	(Dombrowski, Teske and Baker 2018)
Deltaproteobacteria bacterium	455	3	Exoplasmic	RLC06278	Hydrothermal sediments	(Dombrowski, Teske and Baker 2018)
Deltaproteobacteria bacterium	456	3	Exoplasmic	RLB93792	Hydrothermal sediments	(Dombrowski, Teske and Baker 2018)
Deltaproteobacteria bacterium	455	3	Exoplasmic	RLC22838	Hydrothermal sediments	(Dombrowski, Teske and Baker 2018)
Deltaproteobacteria bacterium	414	3	Cytoplasmic	RLC21098	Hydrothermal sediments	(Dombrowski, Teske and Baker 2018)
Deltaproteobacteria bacterium	497	3	Cytoplasmic	RLB22016	Hydrothermal sediments	(Dombrowski, Teske and Baker 2018)
Deltaproteobacteria bacterium	359	3	Cytoplasmic	RLC02598	Hydrothermal sediments	(Dombrowski, Teske and Baker 2018)
Desulfobacteraceae bacterium 4572_187	478	3	Exoplasmic	OQY12990	Hydrothermal sediment	(Dombrowski *et al*. 2017)
Desulfobacteraceae bacterium 4572_89	454	3	Exoplasmic	OQY53460	Hydrothermal sediments	(Dombrowski *et al*. 2017)
Bacteroidetes bacterium	457	3	Exoplasmic	RLD45891	Hydrothermal sediments	(Dombrowski, Teske and Baker 2018)
Bacteroidetes bacterium	447	3	Exoplasmic	RLD65038	Hydrothermal sediments	(Dombrowski, Teske and Baker 2018)
Bacteroidetes bacterium	457	3	Exoplasmic	RLD32997	Hydrothermal sediments	(Dombrowski, Teske and Baker 2018)
Bacteroidetes bacterium	402	1	Exoplasmic	RLD55593	Hydrothermal sediments	(Dombrowski, Teske and Baker 2018)
Bacteroidetes bacterium	469	3	Cytoplasmic	RLD42118	Hydrothermal sediments	(Dombrowski, Teske and Baker 2018)
Bacteroidetes bacterium 4484_249	446	2	Cytoplasmic	OQX80664	Hydrothermal sediments	(Dombrowski *et al*. 2017)
Bacteroidetes bacterium	476	4	Cytoplasmic	RLD38167	Hydrothermal sediments	(Dombrowski, Teske and Baker 2018)
Bacteroidetes bacterium	454	3	Cytoplasmic	RLD75418	Hydrothermal sediments	(Dombrowski, Teske and Baker 2018)
Acidobacteria bacterium	450	3	Cytoplasmic	RLE20106	Hydrothermal sediments	(Dombrowski, Teske and Baker 2018)
Chloroflexi bacterium	453	3	Exoplasmic	RLD03862	Hydrothermal sediments	(Dombrowski, Teske and Baker 2018)
Chloroflexi bacterium	457	3	Exoplasmic	RLD00869	Hydrothermal sediments	(Dombrowski, Teske and Baker 2018)
Chloroflexi bacterium	453	3	Cytoplasmic	RLD11393	Hydrothermal sediments	(Dombrowski, Teske and Baker 2018)
Bacterium	453	3	Cytoplasmic	RKZ14043	Hydrothermal sediments	(Dombrowski, Teske and Baker 2018)
Bacterium	448	3	Cytoplasmic	RKZ19839	Hydrothermal sediments	(Dombrowski, Teske and Baker 2018)
Candidate division KSB1 bacterium	457	3	Exoplasmic	RKY76530	Hydrothermal sediments	(Dombrowski, Teske and Baker 2018)
Candidate division KSB1 bacterium 4572_119	417	1	Exoplasmic	OQX94610	Hydrothermal sediments	(Dombrowski *et al*. 2017)
Candidate division KSB1 bacterium 4484_87	458	3	Exoplasmic	OQX85480	Hydrothermal sediments	(Dombrowski *et al*. 2017)
Candidate division Zixibacteria bacterium	501	3	Exoplasmic	RKX26209	Hydrothermal sediments	(Dombrowski, Teske and Baker 2018)
Candidate division Zixibacteria bacterium	461	3	Cytoplasmic	RKX27199	Hydrothermal sediments	(Dombrowski, Teske and Baker 2018)
Candidatus Aminicenantes bacterium 4484_214	511	3	Cytoplasmic	OQX52307	Hydrothermal sediments	(Dombrowski *et al*. 2017)
Candidatus Aminicenantes bacterium	469	3	Cytoplasmic	RLE02852	Hydrothermal sediments	(Dombrowski, Teske and Baker 2018)
Candidatus Omnitrophica bacterium	389	1	Exoplasmic	RKY41132	Hydrothermal sediments	(Dombrowski, Teske and Baker 2018)
Deltaproteobacteria bacterium	463	3	Exoplasmic	PLX41189	Perchlorate-reducing communities	(Barnum *et al*. 2018)
Salinivirgaceae bacterium	497	4	Exoplasmic	PLX17815	Perchlorate-reducing communities	(Barnum *et al*. 2018)
Marinilabiliales bacterium	456	3	Exoplasmic	PLW95329	Perchlorate-reducing communities	(Barnum *et al*. 2018)
Marinilabiliales bacterium	446	3	Exoplasmic	PLW99613	Perchlorate-reducing communities	(Barnum *et al*. 2018)
Marinilabiliales bacterium	455	3	Cytoplasmic	PLW92978	Perchlorate-reducing communities	(Barnum *et al*. 2018)
Marinilabiliales bacterium	454	3	Cytoplasmic	PLX09622	Perchlorate-reducing communities	(Barnum *et al*. 2018)
Marinilabiliales bacterium	458	3	Cytoplasmic	PLX19442	Perchlorate-reducing communities	(Barnum *et al*. 2018)
Marinilabiliales bacterium	452	3	Cytoplasmic	PLX02242	Perchlorate-reducing communities	(Barnum *et al*. 2018)
Bacteroidetes bacterium GWE2_32_14	432	2	Exoplasmic	OFX85901	Aquifers	(Anantharaman *et al*. 2016)
Bacteroidetes bacterium GWE2_40_15	462	3	Exoplasmic	OFX81662	Aquifers	(Anantharaman *et al*. 2016)
Candidatus Fischerbacteria bacterium RBG_13_37_8	447	3	Cytoplasmic	OGF65237	Aquifers	(Anantharaman *et al*. 2016)
Desulfobacterales bacterium RIFOXYA12_FULL_46_15	456	3	Cytoplasmic	OGR28476	Aquifers	(Anantharaman *et al*. 2016)
Desulfobacteraceae bacterium	476	3	Exoplasmic	RPI80002	Wetlands	(Martins *et al*. 2018)
Deltaproteobacteria bacterium	468	3	Exoplasmic	RPJ06807	Wetlands	(Martins *et al*. 2018)
Bacteroidales bacterium	454	3	Exoplasmic	RPH31952	Wetlands	(Martins *et al*. 2018)
Bacterium SM23_31	446	3	Cytoplasmic	KPK88368	Estuary sediments	(Baker *et al*. 2015)
Candidate division Zixibacteria bacterium SM23_73_2	441	3	Cytoplasmic	KPL04245	Estuary sediments	(Baker *et al*. 2015)
Latescibacteria bacterium DG_63	453	3	Cytoplasmic	KPJ61247	Estuary sediments	(Baker *et al*. 2015)
Deltaproteobacteria bacterium HGW-Deltaproteobacteria-15	542	3	Exoplasmic	PKN64391	Deep terrestrial subsurface sediments	(Hernsdorf *et al*. 2017)
Candidate division Zixibacteria bacterium HGW-Zixibacteria-1	459	3	Exoplasmic	PKK82132	Deep terrestrial subsurface sediments	(Hernsdorf *et al*. 2017)
Desulfobacteraceae bacterium	489	3	Cytoplasmic	RJR39500	Deep terrestrial subsurface fluids	(Momper *et al*. 2017)
Marinimicrobia bacterium 46_43	453	3	Exoplasmic	KUK91590	Oil Reservoirs	(Hu *et al*. 2016)
Candidatus Korarchaeota archaeon	452	3	Exoplasmic	PMB78244	Hot springs	(Wilkins *et al*. 2018)
Desulfobacterales bacterium S5133MH16	488	3	Exoplasmic	OEU64681	Marine sediments	Unpublished
Candidate division KSB1 bacterium	432	3	Cytoplasmic	RQW00415	- ^b^	Unpublished

a Not available; sequence information provided in Supporting Information

b Not available

Of the 11 pure cultures containing hybrid putative *rdh* in their genomes, eight belong to the Marinilabiliales order within Bacteroidetes, that have been isolated from water or sediment samples in marine environment (Table 1). Among these, three strains belong to the genus *Marinifilum*, Gram-negative facultative anaerobes that can tolerate moderate salt concentrations (Na *et al*. 2009; Ruvira *et al*. 2013; Fu *et al*. 2018). Interestingly, hybrid putative *rdh* genes were also found in the MAGs of uncultured Marinilabiliales obtained from perchlorate-reducing enrichment cultures originating from marine sediments (Barnum *et al*. 2018). These genomes mostly lacked respiratory perchlorate, chlorate, oxygen and sulfur reductases and were proposed to be specialized for the fermentation of dead cells (Barnum *et al*. 2018). These finding indicate an important role of the hybrid putative *rdh* genes in Marinilabiliales members. Another pure culture harbouring the hybrid putative *rdh* in its genome is *Caldithrix abyssi*, a thermophilic anaerobic bacterium isolated from a Mid-Atlantic Ridge hydrothermal vent (Miroshnichenko *et al*. 2003). Calditrichaeota are abundant seabed microbes with genomic potential to degrade detrital proteins through the use of extracellular peptidases (Marshall *et al*. 2017).

Except the MAGs obtained from the marine perchlorate-reducing enrichment cultures (Barnum *et al*. 2018), all other MAGs-containing hybrid putative *rdh* were obtained from harsh environments such as hydrothermal vents (Dombrowski *et al*. 2017; Dombrowski, Teske and Baker 2018), hot springs (Wilkins *et al*. 2018), wetlands with extremely high concentrations of dissolved organic carbon and diverse sulfur species (Martins *et al*. 2018), deep terrestrial environments (Hernsdorf *et al*. 2017; Momper *et al*. 2017), etc (Table 1). Most of the sequences from the MAGs were obtained from hydrothermal vent sediments in Guaymas Basin (Gulf of California) with fluctuating temperature and chemical gradients (Dombrowski *et al*. 2017; Dombrowski, Teske and Baker 2018). The MAGs are mostly from uncultured Bacteriodetes and Deltaproteobacteria, but also from the candidate divisions (Table 1). Members of all these phyla have known/proposed diverse metabolic potential, and may not be restricted to reductive dehalogenation. However, physiological proofs for OHR have only been obtained for deltaproteobacterial members with classic *rdh* gene operon i.e. *rdhA*, *rdhB* and one or more transcriptional regulatory genes (Sanford, Chowdhary and Löffler 2016; Liu and Häggblom 2018).

### Outstanding questions

Genomics and allied technologies have greatly increased the diversity of putative *rdh* genes in recent years, and extended their distribution from contaminated environments to deep subsurface (Table 1), Antarctic soils (Zlamal *et al*. 2017), and even human and animal intestinal tract (Atashgahi *et al*. 2018b). With the expanding availability of the bacterial genomes and increasing application of deep sequencing in diverse environments, much more diverse and likely novel *rdh* genes are expected in future. This brings forward major open questions:

Do the newly discovered genes encode RDases? If they indeed encode RDases, what are their functions? Three roles have been shown for the known RDases: energy conservation by OHR, and facilitated fermentation of organic substrates (e.g. pyruvate, lactate or yeast extract) by reoxidation of respiratory cofactors for membrane-bound RDases, and catabolic reductive dehalogenation for cytoplasmic RDases (Fincker and Spormann 2017). Can the hybrid putative RDases with cytoplasmic C-terminus be involved in catabolic reductive dehalogenation, facilitated fermentation or both? In turn, how are the hybrid putative RDases with exoplasmic C-terminus secreted through the cell membrane in absence of TAT signal peptide?
If indeed involved in reductive dehalogenation, what are the physiological organohalogen substrates of the hybrid putative RDases? The lack of correlation between the *rdh* sequences and their organohalogen substrates has precluded the ability to predict substrates for novel genes, and to test their functionality using the predicted organohalogens.
Why the majority of the environmental hybrid putative *rdh* sequences and *rdh*-containing pure cultures have been obtained from harsh environments? Can it be that their physiological organohalogen substrates are found in these environments?
What are the ecological functions of the microbes containing (the hybrid putative) RDases? Detoxification of organohalogens and thereby securing a hospitable environments for themselves and the nearby organisms? Providing carbon sources for themselves (catabolic RDase) or others (respiratory RDase)?
Can (the hybrid putative) RDases be involved in the production of halogenated bioactive compounds as was shown for biosynthesis of marine bacterial pyrroles mediated by a reductive debrominase that utilizes a redox thiol mechanism (El Gamal *et al*. 2016)? Likewise, can the RDases participate in in the production of halogenated bioactive compounds in Eukaryotes such as sponges that are known to harbour Deltaproteobacteria with *rdh* genes (Wilson *et al*. 2014; Liu *et al*. 2017)?
